# Combined Model of Tumor–Stroma Ratio and Tumor Budding Are Not Associated with Tumor Recurrence or Metastasis in Oral Squamous Cell Carcinoma Patients

**DOI:** 10.3390/diagnostics15151844

**Published:** 2025-07-22

**Authors:** Drago Boščić, Emili Dragaš, Andro Košec, Goran Geber, Čedna Tomasović-Lončarić, Davor Vagić

**Affiliations:** 1 Department of Otorhinolaryngology, University Hospital Dubrava, Avenija Gojka Šuška 6, 10000 Zagreb, Croatia; dboscic1@gmail.com; 2 School of Medicine, University of Zagreb, 10000 Zagreb, Croatia; emili.dragas1@gmail.com (E.D.); davorvagic1@gmail.com (D.V.); 3 Department of Otorhinolaryngology and Head and Neck Surgery, University Hospital Center Sestre Milosrdnice, Vinogradska Cesta 29, 10000 Zagreb, Croatia; goran.geber1@gmail.com; 4 Department of Pathology, University Hospital Dubrava, Avenija Gojka Šuška 6, 10000 Zagreb, Croatia; ctomasov@gmail.com; 5 School of Medicine, Catholic University of Croatia, 10000 Zagreb, Croatia

**Keywords:** oral squamous cell carcinoma, tumor budding, histopathology, prognosis, tumor–stroma ratio

## Abstract

Histopathological grading of oral squamous cell carcinoma is currently based on differentiation of cells, while additional histological parameters, such as the tumor–stroma ratio (TSR), tumor budding (TB), or the combined TSR/tumor budding model could better assess tumor biological behavior and monitoring of patients. **Background/Objectives**: To integrate risk factors associated with tumor progression: the TSR, TB and TSR/tumor budding model, whose prognostic significance in oral cancer has not yet been evaluated. **Methods**: An observational cohort retrospective study assembled according to STROBE guidelines on histological materials from 196 patients with invasive squamous cell carcinoma of the oral cavity. The goal of the analysis was to evaluate the association between the tumor stroma ratio, tumor budding, and the combined model of TSR/TB with the clinical and pathologic features of patients with squamous cell carcinoma of the oral cavity and to determine the prognostic value of this model in relation to disease-free survival (DFS) **Results**: The analysis did not show that the tumor stroma ratio (TSR), tumor budding, and the combined model of TSR/tumor budding were statistically significantly associated with the occurrence of metastatic disease at the start of treatment or during postoperative follow-up, but confirmed the value of depth-of-invasion (DOI) as a negative prognostic factor (HR 15.3, *p* < 0.001). **Conclusions**: The TSR, TB, and the combined TSR/TB model were not found to be statistically significant predictors for the disease progression in the Cox regression survival analysis but were found to have a significant correlation with known negative prognostic factors: DOI, neural invasion, and T category.

## 1. Introduction

Squamous cell carcinoma of the oral cavity is highly prevalent, with over 300,000 cases occurring globally every year. These tumors carry a significant burden, with a high mortality rate of 48% [1]. Among different histological subtypes, squamous cell carcinoma (OSCC) is the most common, accounting for over 90% of cases [2]. Given the high incidence and mortality rate, there is a growing demand for reliable factors associated with disease prognosis and enabling treatment tailoring. Apart from the TNM system of cancer staging that takes into account the size of the primary tumor (T), involvement of locoregional lymph nodes (N), and distant metastases (M), in the 8th edition of the American Joint Committee of Cancer, depth of tumor invasion (DOI) in the T stage and extra-capsular spread in the N stage were also taken into consideration [3]. Histopathological grading is currently based on differentiation of cells, which alone does not strongly correlate with prognosis, while additional histological parameters, such as the tumor–stroma ratio (TSR) and tumor budding (TB), or rather the combined TSR/tumor budding model, could better assess biological behavior and enable improved therapeutic approaches and monitoring of patients with oral squamous cell carcinoma [4]. The tumor–stroma ratio (TSR) is a method for assessing the proportion of the tumor stroma within the tumor on routine histological slides. According to the results of the meta-analysis by Wu et al. [5], solid tumors with a higher proportion of stroma are associated with poor overall survival (OS) and disease-free survival (DFS). This meta-analysis was focused on solid tumors in general, further analyzing some cancer types (colorectal carcinoma, hepatocellular carcinoma, ovarian cancer, and nasopharyngeal carcinoma, etc.). A low TSR (“stroma-rich tumors”) was associated with a decreased OS as well as DFS in CRC and higher stroma content is significantly associated with clinical stage, advanced invasion depth, and positive lymph node metastases [5]. A systematic review and meta-analysis by Almangush et al. studied the TSR in head and neck cancers. The meta-analysis showed that in oral carcinoma, TSR has a correlation with OS and DFS [6]. Tumor budding (TB) is defined as the presence of isolated tumor cells or small cell clusters (up to 5 cells) within the tumor stroma, located near or at the invasive tumor front. It represents an epithelial–mesenchymal transition (EMT) in which cells lose intercellular contact as well as contact with the extracellular matrix, gaining migratory/invasive potential. TB is recognized as an additional prognostic factor for colorectal cancer (CRC) according to the UICC (Union for International Cancer Control) and a potential prognostic factor for early-stage cancer according to the ESMO (European Society for Medical Oncology) consensus guidelines [7,8]. It has also been studied in lung and breast cancer [9,10,11]. Several studies investigated its role in OSSC, demonstrating the association of TB and EMT and proposing that TB is a good tissue-based biomarker for prognosis due to its correlation with OS and DFS [12,13,14,15].

Therefore, the aim of this study was to integrate risk factors associated with tumor progression, namely the tumor–stroma ratio (TSR), tumor budding, and the TSR/tumor budding model, whose prognostic significance in oral cancer has not yet been fully evaluated. The integrated model represents a potential prognostic factor for improving risk stratification, with an assessment that is simple, rapid, and reproducible, and could be incorporated into routine histopathological diagnostics.

## 2. Materials and Methods

This observational cohort retrospective study was assembled according to STROBE guidelines and conducted in the University hospital Dubrava in Zagreb, Croatia. Tissue samples of primary squamous cell carcinoma of the oral cavity were used, diagnosed after surgical excision from the period between 2011 and 2017 and stored in the archives of the Clinical Institute for Pathology and Cytology at Clinical Hospital Dubrava. Approval from the hospital IRB was obtained, according to the Helsinki Declaration. The study included histological materials from patients with confirmed invasive squamous cell carcinoma of the oral cavity who had not previously received chemotherapy or radiotherapy, had no other malignant disease at the time of diagnosis, and were monitored for at least five years after surgery. Patients were followed up postoperatively, and their oncological status was recorded, with the main outcome being the time to disease-free survival. At the time of diagnosis, patients had no other clinically detectable malignant disease. Patients were selected from the specified period to ensure a minimum five-year follow-up. A ten-year follow-up, considering the retrospective nature of the study, would require cases diagnosed before 2011, whose biopsy materials are no longer available in the archive due to the expiration of the legally mandated storage period. In total, 196 samples were analyzed. Clinical data (age, sex, tumor localization, pathohistological characteristics: tumor size, degree of differentiation, perineural invasion, lymphovascular invasion, depth of invasion, lymph node metastases, pTNM stage) and disease progression data were obtained from medical records in the electronic database of University Hospital Dubrava (BIS) and the Clinical Institute for Pathology and Cytology (Issa).

**Figure 1:**
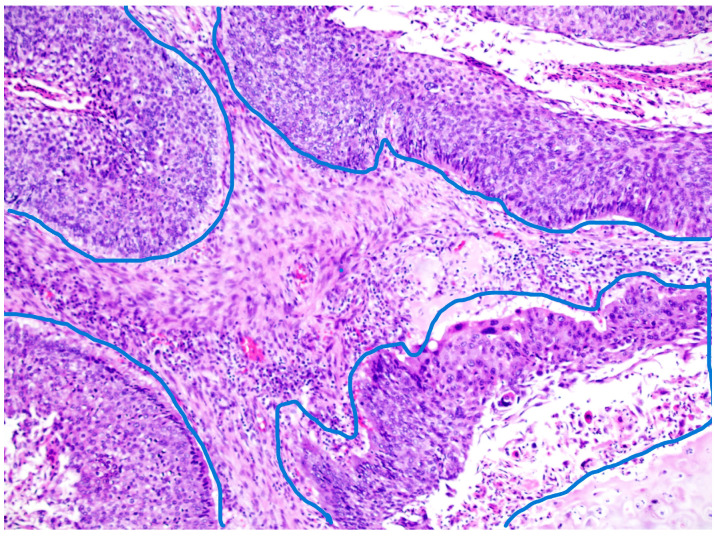
Tumor–stroma ratio (TSR)—tumor area encircled by blue line, >50% stroma content, hematoxylin and eosin stain, magnification 10×.

**Figure 2:**
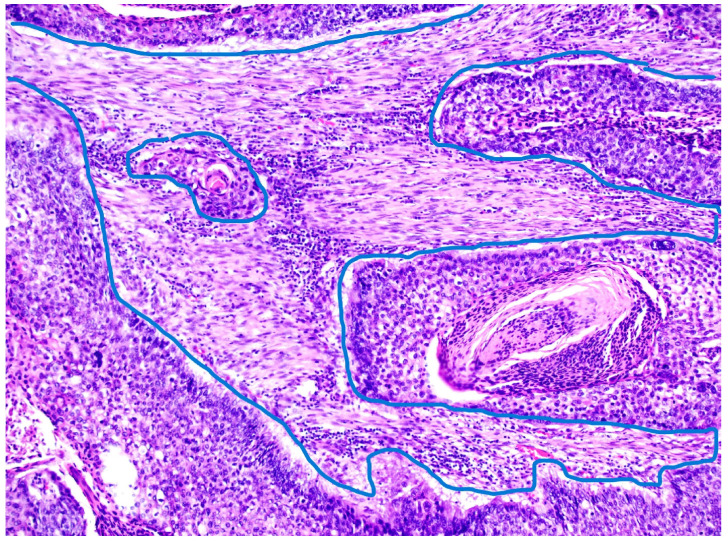
Tumor–stroma ratio (TSR)—tumor area encircled by blue line, <50% stroma content, hematoxylin and eosin stain, magnification 10×.

**Figure 3:**
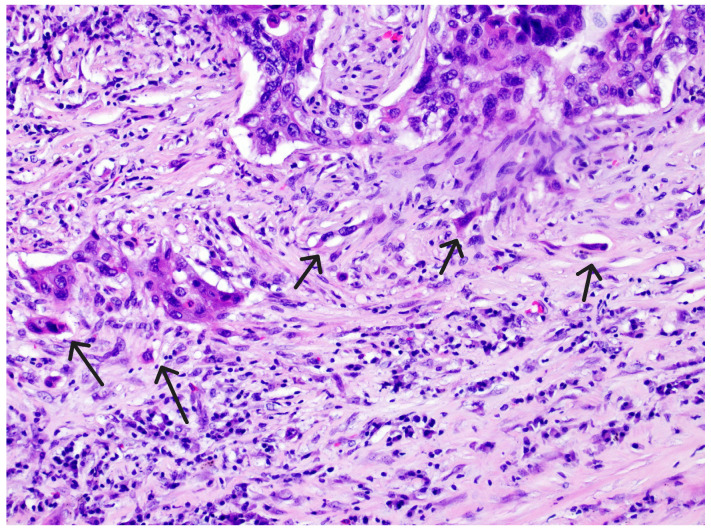
Tumor budding (TB)—tumor buds (arrows) defined as single cancer cells and/or clusters of ≤4 cells, hematoxylin and eosin stain, magnification 20×.

**Figure 4:**
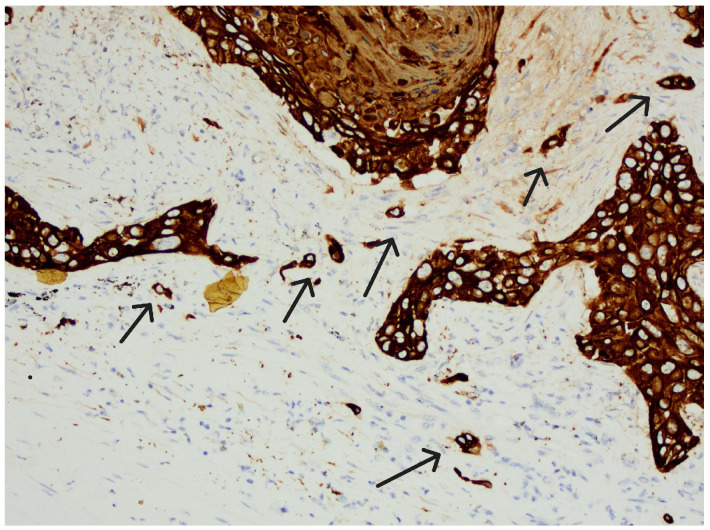
Tumor budding (TB)—tumor buds (arrows), positive immunohistochemically for CKAE1/AE3, >5 TB, magnification 20×.

The tumor stroma ratio (TSR) was determined according to the method proposed by van Pelt [16]. The archived tissue samples were re-examined to select a representative sample from the site of deepest invasion with the highest amount of tumor stroma for each individual case. H&E-stained tissue sections of OSCC were analyzed at the lowest magnification (4×) to select the area with the highest amount of tumor stroma. TSR was then assessed within single field of view at 10× magnification with both stroma and tumor cells included within the field, ensuring that tumor cells are present on all four sides of the field. Necrosis and entrapped non tumor tissues, like muscle or salivary glands, were not considered part of tumor stroma. The analyzed field of view, excluding any tissue that was disregarded if present, was considered 100%, and the proportion of tumor stroma in relation to neoplastic cells was estimated in 10% increments. Tumors with a TSR ≥ 50% were classified as “stroma-rich tumors,” while those with a TSR < 50% were classified as “stroma-poor tumors.” (Figure 1 and Figure 2). Tumor budding (TB), defined as the presence of single tumor cells or small clusters of up to four cells (≤4) at the invasive front, was firstly assessed by selecting the H&E-stained tissue section with the greatest degree of budding at the invasive font and then scanning several fields at 10× magnification to identify the hotspot with highest number of tumor buds at the invasive front. Finally, tumor buds were counted in the determined hotspot within a single field of view at 20× magnification. A cutoff value of five buds was established, distinguishing cases with <5 buds from those with ≥5 buds per field of view [17]. The obtained parameters, including the tumor–stroma ratio (TSR) and tumor budding, were then further combined and categorized into three risk groups. Tumors with a TSR < 50% and <5 tumor buds were classified as “low-risk” carcinomas. Those with a TSR < 50% and ≥5 tumor buds, as well as those with a TSR ≥ 50% and <5 tumor buds, were categorized as “intermediate-risk” carcinomas. Finally, tumors with a TSR ≥ 50% and ≥5 tumor buds were considered “high-risk” carcinomas. (Figure 3 and Figure 4).

Disease progression was defined as the occurrence of local recurrence, loco-regional metastases, or distant metastases.

A power analysis was conducted based on the average number of tumor buds as a variable with a cutoff value of 5 and a standard deviation of 1, along with four possible categories of immunohistochemical results depending on the percentage of tumor stroma ratio in relation to the cutoff value of 50%. Alpha was set at 0.05, and the power of the test was 80%. The power analysis determined the minimum required sample size to establish statistical significance to be 126 patients.

In the statistical analysis of the data, the initial time point was sample collection during the surgical procedure, while the endpoint was the occurrence of malignant disease recurrence in patients (local recurrence, locoregional metastases, or distant metastases), or the end of follow-up, designated as a censored time point. The goal of the data analysis was to evaluate the association between the tumor stroma ratio (TSR), tumor budding (TB), and the combined model of TSR/TB with the clinicopathological features of patients with squamous cell carcinoma of the oral cavity and to determine the prognostic value of this model in relation to disease-free survival (DFS), where disease recurrence is defined as the occurrence of cytologically and histopathologically confirmed regional metastases. The presence of TSR, TB, and the combined TSR/TB model was compared with the histopathological characteristics of the tumors: tumor size, depth of tumor invasion, tumor localization, grade, pTNM categories, lymphatic invasion, angioinvasion, extranodal extension, patient age, and sex.

The normality of data distribution was checked using the Smirnov–Kolmogorov test, and depending on the results, non-parametric tests were used along with appropriate representations of continuous values, since all variables did not show a normal distribution. The outcome of the follow-up (recurrence present or absent) was determined using census analysis. Given the large number of variables, tests that could account for unfavorable ratios between the number of subjects and variables were employed. To reduce the potential negative impact of a small sample size on testing a large number of variables, Kruskal–Wallis tests were used, along with binary logistic regression, which is less sensitive to a large number of variables and allows for the establishment of causal relationships between dependent and tested variables.

For survival analysis, multivariate Cox regression analysis was employed, with time to recurrence as the follow-up time in months. As a confirmatory method, principal component analysis (PCA) was performed with Oblimin rotation to identify groups of variables that appear together and to confirm the results of the multivariate regression analysis. Variables were compared using the Spearman’s rho correlation coefficient. All statistical tests were two-tailed. A *p*-value of less than or equal to 0.05 was considered statistically significant. Data analysis was performed using MedCalc software (Version 11.2.1^©^ 1993–2010, MedCalc Software bvba, Software, Broekstraat 52, 9030 Mariakerke, Belgium) and SPSS software (Version 22.0, released in 2013, IBM SPSS Statistics for Windows, IBM Corp., Armonk, NY, USA).

## 3. Results

### 3.1. Sample Features

The study included 196 participants who were surgically treated for histopathologically confirmed squamous cell carcinoma at KB Dubrava from 1 January 2011, to 31 December 2017. There were 151 male and 45 female participants, resulting in a male-to-female ratio of 3.36. The average age was 63.13 years, with a range from 36 to 92 years and a standard deviation of 10.99 years. The distribution of demographic and histopathological variables is shown in Table 1.

### 3.2. Analysis of the Follow-Up Interval and the Known Natural History of the Disease—Sample Validation

**Figure 5:**
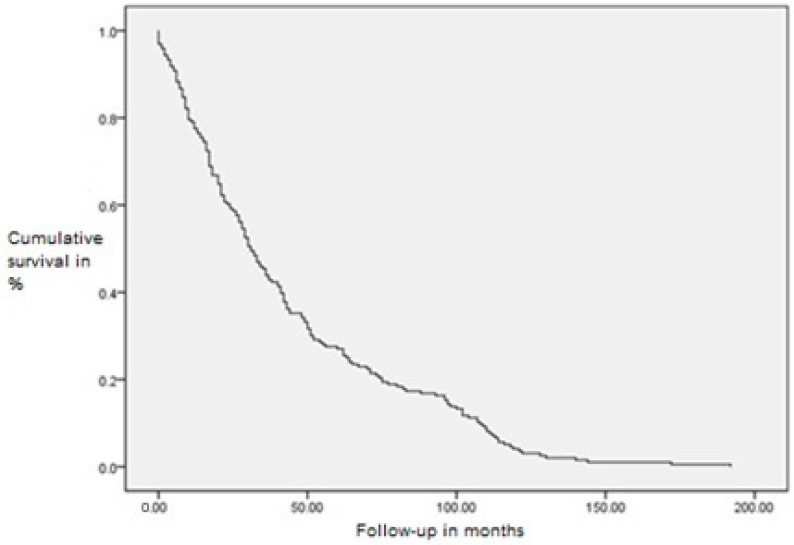
Kaplan–Meier survival curve of follow-up with cumulative survival.

The average follow-up duration for patients, expressed as the mean and standard deviation, was 44.9 ± 38.7 months. Due to the large standard deviation, the median is a more precise measure of follow-up duration, which was 34 months. Detailed descriptive statistics for patient follow-up are shown in the histogram of follow-up duration until disease occurrence, obtained from the Kaplan–Meier survival curve (Figure 5).

The completeness of follow-up is expressed as censored or complete. Complete follow-up refers to cases where the critical event, in this case, recurrence or the occurrence of regional disease in patients, was recorded within the minimal 5-year follow-up period. Censored follow-up indicates cases where the critical event did not occur within that time. In the group of participants, there were 49 critical events and 147 censored cases. An increase in hazard is observed, inversely proportional to survival.

**Figure 6:**
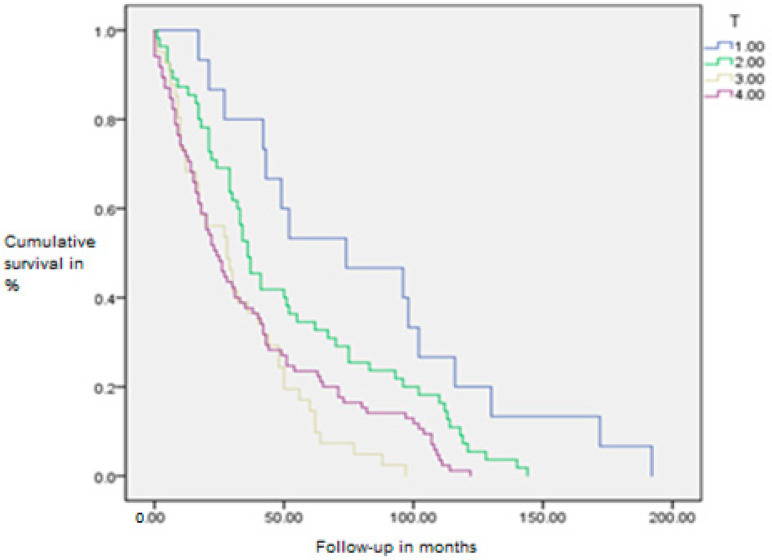
Kaplan–Meier survival curve by T category of the disease.

**Figure 7:**
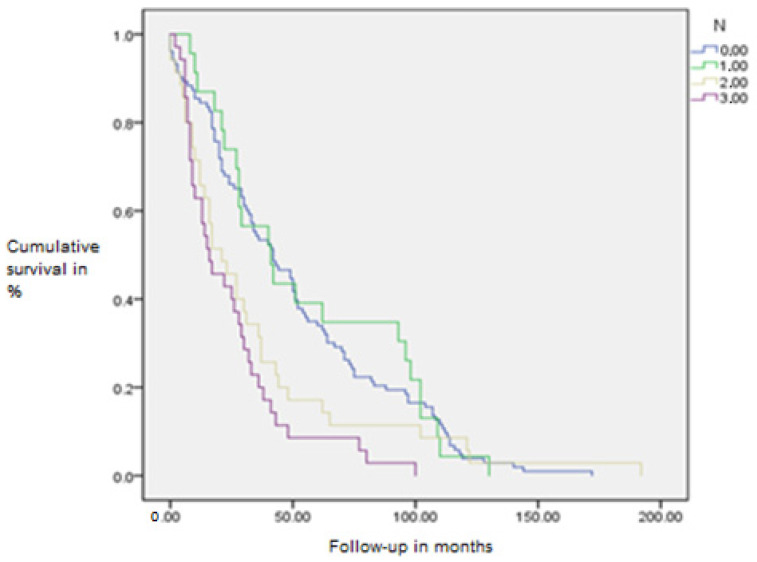
Kaplan–Meier survival curve by N stage of the disease.

There is a noticeable lower survival rate in patients with higher T categories and N stages of the disease, which is consistent with known data and validates this statistical model on the dataset (Mantel-Cox log-rank test, *p* = 0.007 and *p* = 0.001, respectively) (Figure 6 and Figure 7).

### 3.3. Assessment of the Prognostic Value of the Proposed TSR/Tumor Budding Ratio, TB and TSR

A statistical analysis was performed to assess the association between tumor stroma ratio (TSR), tumor budding, and the combined model of TSR/tumor budding in patients with oral squamous cell carcinoma using a binary logistic regression model, with the presence of metastatic disease at the start of treatment and the occurrence of metastatic disease during postoperative follow-up as dependent variables. No statistically significant associations were found between the occurrence of metastatic disease at the start of treatment and its subsequent appearance during follow-up.

A survival analysis was also performed on the participants, with time to the critical event as the endpoint, using multivariate Cox regression. This analysis also did not show that the tumor stroma ratio (TSR), tumor budding, and the combined model of TSR/tumor budding were statistically significantly associated with the occurrence of metastatic disease at the start of treatment or during postoperative follow-up, but confirmed the value of DOI as a negative prognostic factor (HR 15.3 (CI9513.07–16.52), *p* < 0.001).

As it is well known that increasing T and N categories, tumor invasion depth, and the presence of extranodal extension, neural, and angioinvasion negatively affect survival, the role of the tumor stroma ratio (TSR), tumor budding, and the combined model of TSR/tumor budding on disease prognosis was further analyzed using ancillary statistical analysis.

The Kruskal–Wallis test analysis for independent samples showed that the tumor stroma ratio (TSR) (*p* = 0.041, chi-square, *p* = 0.05) and the combined model of TSR/tumor budding (Kruskal–Wallis, *p* = 0.002 and chi-square, *p* = 0.016) were statistically significantly associated with increasing T categories of the disease.

The tumor stroma ratio showed a statistically significant association with the presence of neural invasion (Kruskal–Wallis, *p* = 0.05, chi-square *p* = 0.034).

The N category of the disease, the presence of extranodal extension, tumor grade, and the presence of angioinvasion did not show a statistically significant association with the predictive variables of the test.

Since T1 and T2 tumors made up 70 samples, the majority of samples were taken from advanced cancer patients, we performed an ancillary analysis. Our binary logistic regression model (with early cancer (T1 and T2) designated as 0 and advanced (T3 and T4) designated as 1), showed statistical significance for advanced tumors as follows: DOI (*p* = 0.001, OR 71.615), ENE (*p* = 0.001, OR 13.505), neural invasion (*p* = 0.001, OR 22.031), angioinvasion (*p* = 0.023, OR 5.167), combined TSR/TB score (*p* = 0.021), and disease free survival (*p* = 0.05, OR 3.797). This would indicate that the progression from early to deeper-invading tumors may yet be significantly associated with TSR and TB indices.

The Spearman’s rho correlation analysis showed a statistically significant correlation between the tumor stroma ratio and tumor invasion depth (correlation coefficient 0.197, *p* = 0.006), the presence of neural invasion (correlation coefficient 0.141, *p* = 0.049), and the combined score and tumor invasion depth (correlation coefficient 0.182, *p* = 0.011).

### 3.4. Principal Component Analysis of the Sample Variables as a Confirmatory Analysis and Sampling Bias Correction

In order to confirm the results and show that there were no confounding variables, a principal component analysis was performed using all the examined variables. The analysis identified 10 components that cumulatively explain 86.6% of the variance, with the first three components explaining 46.626% of the variance (Table 2). This method is used to identify clusters of variables associated together by rotating the dataset and allowing for more freedom than traditional statistical tests oriented at a single dependent variable and are especially useful in datasets with a large number of variables. The scree plot showed that the first two components have eigenvalues > 2 and can be considered significant principal components, which translates into significant associations between variables sorted together in the component groups. To interpret each principal component, we examined the magnitude and direction of the coefficients for the original variables. The larger the absolute value of the coefficient, the more important the corresponding variable is in calculating the component. The associations were considered significantly associated if their coefficient exceeded 0.2.

The correlation coefficients of the first and second components show that the values of the variables TSR, TB, and the combined score significantly contribute to the variance of the first three components, along with the most important negative prognostic factors such as neural and angioinvasion, T category, tumor invasion depth, presence of extranodal extension, and metastatic disease. They also show a significant negative correlation with the duration of follow-up and sex.

## 4. Discussion

In our study, the aim was to analyze the two known prognostic factors associated with tumor progression, TSR and TB, as well as the TSR/tumor budding model, which has not yet been investigated in routine medical practice. Upon statistical analysis, TSR, TB and the combined model did not show significant association with the presence of disease recurrence either at the beginning of treatment or during postoperative follow-up as well as with disease free survival. They did however show a negative correlation with the duration of patient follow-up which may indicate shorter survival in patients with higher values of these variables. Also, all three of the parameters, TSR, TB and TSR/tumor budding, showed a significant correlation with well-established negative prognostic factors such as T category, depth of invasion, and perineural invasion. All of these factors were described as promising prognostic markers in the early stages of oral tongue cancer in the systematic review and meta-analysis by Elseragy and colleagues [15,18,19]. Most studies of TSR in various malignant epithelial tumors have shown a significant association between stroma rich tumors and invasiveness, lymph node metastases, clinical stage, and consequently, decreased OS and DFS [5,6]. Only a few studies found no significant association between TRS and poor disease outcome indicators, mainly due to discrepancies in TSR assessment [6]. In studies that have confirmed the value of TSR as a prognostic indicator, the assessment method proposed by van Pelt was used, which proved to be reproducible, with good interobserver agreement, minimizing the impact of tumor heterogeneity, and easy to assess on routinely stained hematoxylin and eosin (HE) slides [16]. Tumor budding is considered an adverse prognostic factor in various carcinomas [9,10,11]. It has been most extensively studied in colorectal cancer and has been included in many international clinical guidelines and protocols [20,21,22]. Although studies of TB in OSCC have not been so numerous and systematic as in CRC, published results demonstrated a significant association with survival, recurrence of disease, lymph node metastases, tumor size, and histological features like pattern of invasion and DOI [12,13,14,15]. Worst pattern of invasion (WPOI) analysis is also included in protocols for the assessment of oral squamous cell carcinoma. WPOI has been validated on multivariate analysis in OSCC in low stage tumors where the presence of WPOI type 4 and 5 is associated with larger tumor size, poor differentiation, perineural invasion (PNI), greater depth of invasion (DOI), and higher incidence of nodal metastasis. Among the 5 patterns observed, WPOI-5 is the most relevant as a prognostic indicator for OSCC. Given that WPOI-5 includes both perineural and lymphovascular dissemination, according to the CAP protocol for oral squamous cell carcinoma, WPOI is somewhat redundant and can be considered optional for reporting purposes. There are some similarities between tumor budding and WPOI. WPOI-4 and 5 are defined as invasive growth in small islands of up to 15 cells but outside the tumor perimeter, while tumor budding is defined as tumor growth in small clusters of up to 4 cells either at the tumor invasive front or as intratumoral budding. TB is probably more common in WPOI-4 and 5 where there is a clear tendency to grow in discohesive clusters, which does not exclude TB also occurring in WPOI-1, 2 or 3, defined by growth either by a pushing front, a finger-like pushing front, or large (>15 cells) tumor islands within the tumor perimeter. Whether tumor budding has any prognostic significance in such cases has probably not been specifically studied [23,24]. Interestingly, our study did not show a direct association of TB with survival, as it did in certain previously mentioned studies [14]. Unlike TB in colorectal cancer (CRC) for which the agreement on an international, evidence-based standardized scoring system has been reached, there is no standardized method for tumor budding assessment in OSCC. In this study, the assessment area and cut-off values for TB were determined for simplicity according to the method of TB reporting in CRC recommended by the International Tumor Budding Consensus Conference (ITBCC), although the normalization factor to determine the tumor bud count per 0.785 mm^2^ was not used [17]. We believed that the correction factor itself would not have an impact on the results within the observed group, especially since no standardized assessment method was used to assess TB in OSCC in other studies that showed an association with disease recurrence, lymph node metastases, and survival [12,13,14,15]. The omission of the normalization factor could certainly affect the results of the TB assessment as well as the TSR/TB method, which only indicates the need to conduct wider multicenter studies to standardize the method of assessing tumor budding and determine adequate values for OSCC, before it is implemented in routine practice. Further investigations should also examine appropriate cut-off values for TB in OSCC. The question is whether a cut-off value of <5 and ≥5 buds is appropriate for all cancer types. Considering the large patient sample size in our study (*N* = 196) and the clear pathophysiological basis of our hypothesis, the question arises as to why the results differ from previously published research. The discrepancy may be due to differences in design, as some of the studies focused on early-stage tumors only. The present study is made up of all tumor stages. The meta-analysis by Elseragy et al. focused specifically on the early stages of cancer while others analyzed TB/TSR in various clinical stages [6,12,13,14,15,18,19]. Since our sample was composed of 70 early-stage and 126 advanced tumors, an ancillary analysis showed that the combined TSR/TB score is associated with the progression from early to deeper-invading tumors, indicating that additional analyses should be performed in advanced tumors to confirm this observation.

There are limited data in the literature on the combined model but a study published by Dourado et al. demonstrated its prognostic potential in OSCC. They evaluated all three parameters, highlighting their prognostic potential, specifically of the combined model in risk stratification [25], supporting our hypothesis that a combined model could serve as an even more effective prognostic tool in OSCC pathology analysis. When patients were stratified into early and advanced stage groups, TSR, TSB, and TSR/TB were significantly associated with cancer-specific survival, while TSR and TSR/TB but not TB, were significantly associated with disease-free survival in early stage tumors. In advanced stage tumors TSR and TSR/TB were significantly associated with cancer-specific survival, while no variable was associated with disease-free survival. These results, as well as the results obtained in our study after additional analyses, justify the validation of TRS, TB, and TSR/TB in stage-stratified cohorts.

It is known that tumor cells at the invasive front, where cancer cells face stromal cells and ECM, undergo epithelial–mesenchymal transition (EMT), a process which allows cells to detach and gain mobility and invasive ability [26]. TB basically represents cells undergoing EMT and has been significantly associated with high amount of tumor stroma [6]. Therefore, we believed that by combining these two factors (TB and TSR) we could obtain a more sensitive prognostic indicator. Although our data did not prove a direct association with the presence of lymph node metastases, locoregional recurrence, or survival, it did, however, show a significant association of TSR, TB, and TSR/tumor budding and known negative prognostic factors (T category, depth of invasion, and perineurial invasion), guiding us to a conclusion that these parameters may still reflect poor prognosis through their association with these adverse prognostic factors. Depth of invasion (DOI) was proven to be a valuable prognostic marker due to its association with mortality, locoregional recurrence, and occult lymph node metastasis [27,28]. Therefore, DOI was incorporated into the T staging of the TNM in its 8th edition in 2017, by the American Joint Committee on Cancer Control (AJCC) [29]. With no significant improvement in patient survival outcomes of oral cancers and more data demonstrating the need for early evaluation of tumor behavior and prognosis, there is a need for other morphological parameters, such as TB and TSR, that could enhance prognostic accuracy. The decision on further treatment is currently based on pathological TNM staging and WHO grading, which does not offer ideal prognostic modelling, as 18% of early-stage tumors are associated with cancer-related mortality [18,19]. Considering the well-established pathophysiological basis of our hypothesis and the association of the combined model with known negative prognostic factors, this paper underscores the need for further exploration of these parameters, such as the combined TSR/tumor budding model, to refine diagnostic and prognostic approaches for oral squamous cell carcinoma and the possible addition of TB and TSR analysis in routine medical practice. It additionally emphasizes the importance of already proven prognostic histopathological parameters, such as DOI, in oral cancer prognosis [27,28,29].

The limitations of this study are differences in confirming the predictive value for survival and metastatic progression compared to previous studies, albeit on a large patient sample, that limit detailed analysis. In order to perform a comprehensive analysis comparable with studies on early-stage cancer, the authors would have needed to perform a separate analysis, but our overall sample size did not allow for a meaningful analysis due to low statistical power. Although numerous studies have suggested promising prognostic factors related to these parameters, our findings reinforce that DOI should still be considered the primary histologic prognostic factor in oral carcinoma.

## 5. Conclusions

The TSR, TB, and the combined TSR/TB model were not found to be statistically significant predictors for the disease progression in the Cox regression survival analysis but were found to have a significant correlation with known negative prognostic factors: DOI, neural invasion, and T category.

## Figures and Tables

**Table 1:** Descriptive statistics of variables in the sample.

All participants	*n* = 196
Male	*n* = 151
Female	*n* = 45
Age in years	
Average ± SD	63.13 ± 10.99
Median	61
Values of histopathological variables	
Grade 1	66 (33.7%)
Grade 2	95 (48.5%)
Grade 3	31 (15.8%)
Grade 4	4 (2%)
Extranodal extension	45 (22.9%)
Angioinvasion	31 (15.8%)
Perineural invasion	91 (46.4%)
Average tumor stroma ratio	39.6
Tumor budding	
Grade I (<5)	130 (66.3%)
Grade II (>5)	66 (33.7%)
Combined score	
Low risk	85 (43.4%)
Intermediate risk	74 (37.8%)
High risk	37 (18.9%)
Average depth of tumor invasion (mm)	15.09
Average tumor diameter (mm)	32.1
Number of patients with metastatic disease at the start of treatment	93 (47.4%)
Number of patients with the occurrence of metastatic disease during follow-up	49 (25%)
TNM categories	
pT1	15 (7.6%)
pT2	55 (28.1%)
pT3	41 (20.9%)
pT4	85 (43.4%)
N0	103 (55.6%)
N1	23 (11.7%)
N2	35 (17.9%)
N3	35 (17.9%)
Localization	
Retromolar	37 (18.9%)
Manidibular gingiva	49 (25%)
Sublingual	59 (30.1%)
Tongue	29 (14.8%)
Maxillary gingiva	9 (4.6%)
Hard palate	13 (6.6%)
Average follow-up in months	44.9

**Table 2:** Display of correlation coefficients in the matrix of the first three principal components.

	Component					
	1	2	3	4	5	6
Age	−0.073	−0.069	−0.433	0.615	−0.105	−0.384
Sex	−0.254	0.019	−0.072	0.703	0.291	−0.074
Localization	0.056	−0.107	−0.126	0.250	−0.490	0.603
T	0.668	0.174	−0.499	−0.068	0.058	0.058
Tumor diameter	0.631	0.104	−0.571	−0.027	0.168	0.105
DOI	0.702	0.159	−0.481	−0.009	0.015	0.140
N	0.765	−0.402	0.378	0.206	0.044	0.037
ENE	0.673	−0.324	0.281	0.238	0.029	0.222
Metastases	0.660	−0.390	0.433	0.197	0.043	−0.036
Gradus	0.280	0.063	0.136	−0.129	−0.573	−0.351
Neural invasion	0.560	0.017	−0.188	−0.137	0.103	−0.332
Angioinvasion	0.464	−0.207	0.205	−0.186	0.094	−0.293
TSR	0.285	0.595	0.261	0.089	0.235	−0.104
TB	0.156	0.722	0.263	0.103	−0.139	0.125
Combined score	0.225	0.826	0.353	0.173	0.064	0.053
Recurrence	0.348	0.009	0.058	−0.245	0.243	0.214
Follow up	−0.476	−0.193	0.040	−0.069	0.479	0.198

## Data Availability

Data available from the corresponding author upon reasonable request.
